# Anti-Inflammatory Functions of Protein C Require RAGE and ICAM-1 in a Stimulus-Dependent Manner

**DOI:** 10.1155/2014/743678

**Published:** 2014-05-04

**Authors:** Natascha Braach, Kirsten Buschmann, Johanna Pflaum, Hannes Hudalla, Lutz Koch, Eduard Ryschich, Johannes Poeschl, David Frommhold

**Affiliations:** ^1^Department of Neonatology, Ruprecht-Karls-University Hospital Heidelberg, 69120 Heidelberg, Germany; ^2^Department of Surgery, Ruprecht-Karls-University Hospital Heidelberg, 69120 Heidelberg, Germany

## Abstract

By binding *β*_2_-integrins both ICAM-1 and the receptor for advanced glycation end products (RAGE) mediate leukocyte recruitment in a stimulus-dependent manner. Using different inflammatory mouse models we investigated how RAGE and ICAM-1 are involved in anti-inflammatory functions of protein C (PC; Ceprotin, 100 U/kg). We found that, depending on the stimulus, RAGE and ICAM-1 are cooperatively involved in PC-induced inhibition of leukocyte recruitment in cremaster models of inflammation. During short-term proinflammatory stimulation (trauma, fMLP, and CXCL1), ICAM-1 is more important for mediation of anti-inflammatory effects of PC, whereas RAGE plays a major role after longer proinflammatory stimulation (TNF**α**). In contrast to WT and *Icam-1^−/−^* mice, PC had no effect on bronchoalveolar neutrophil emigration in *RAGE^−/−^* mice during LPS-induced acute lung injury, suggesting that RAGE critically mediates PC effects during acute lung inflammation. In parallel, PC treatment effectively blocked leukocyte recruitment and improved survival of WT mice and *Icam-1-*deficient mice in LPS-induced endotoxemia, but failed to do so in *RAGE-*deficient mice. Exploring underlying mechanisms, we found that PC is capable of downregulating intracellular RAGE and extracellular ICAM-1 in endothelial cells. Taken together, our data show that RAGE and ICAM-1 are required for the anti-inflammatory functions of PC.

## 1. Introduction

The vitamin K-dependent serine protease protein C (PC) is activated upon binding of thrombin to thrombomodulin (TM) which is supported by the endothelial protein C receptor (EPCR) [1]. The PC-TM-EPCR complex activates protease activated receptor 1 (*PAR-1*), and as a consequence activated protein C (aPC) elicits potent anti-inflammatory and cytoprotective effects besides its common anticoagulatory properties [1, 2]. Activation of* PAR-1* inhibits NF-*κ*B translocation to the nucleus which results in a reduced production of proinflammatory cytokines and expression of cell adhesion molecules such as intercellular adhesion molecule 1 (ICAM-1) and vascular cell adhesion molecule 1 (VCAM-1) [3] and thereby blocks leukocyte recruitment. The cascade of leukocyte recruitment plays a crucial role in the immune defense during inflammation [4]. The capture of free flowing leukocytes is followed by leukocyte rolling along the endothelial layer, triggering the activation of the *β*_2_-integrins LFA-1 and Mac-1 which interact with different endothelial ligands such as ICAM-1 and RAGE, the receptor for advanced glycation end products [5, 6]. This promotes firm adhesion to the inflamed endothelium and finally leads to leukocyte transmigration [4, 7].

Previous* in vitro* and* in vivo* studies have shown that leukocyte recruitment can be blocked by aPC in various models of inflammation [8, 9]. There is increasing evidence that this holds true for the zymogen protein C [10–12]. We previously demonstrated that protein C concentrate gets sufficiently activated* in vivo*, blocks leukocyte adhesion and transmigration in different mouse models of inflammation, and improves survival during systemic inflammation [11]. Numerous reports stress the anti-inflammatory properties of PC in inflammatory conditions and diseases beyond sepsis [13, 14]; however, the mechanisms of action are only partially uncovered.

The pattern recognition receptor RAGE, a strong activator of the proinflammatory transcription factor NF-*κ*B, serves as a signaling molecule in the innate immune system and is thus involved in various inflammatory diseases [15, 16].* RAGE*-deficient mice showed decreased mortality compared to wild-type mice in mouse models of systemic inflammation [17, 18]. Besides its signaling function, RAGE controls leukocyte recruitment as a direct ligand for the *β*_2_-integrin Mac-1 [5, 6, 19]. Thus, RAGE cooperates with ICAM-1 in mediating leukocyte recruitment during inflammation depending on the stimulus [5, 6].

In this study we raised the question whether RAGE collaborates with ICAM-1 in mediating anti-inflammatory properties of PC, particularly the capacity to block leukocyte recruitment. Therefore, we studied the effect of PC on leukocyte recruitment in *RAGE* ^−/−^ and* Icam-1* ^−/−^ mice using intravital microscopy of cremaster muscle venules in short-term (trauma, fMLP, and CXCL-1) and long-term (TNF-*α*) inflammation models. To increase the clinical relevance we observed leukocyte recruitment and survival after inflammatory stimulation with LPS during LPS-induced acute lung injury or LPS-induced endotoxemia in these groups. In addition, we analyzed the expression of adhesion molecules in response to PC treatment.

## 2. Materials and Methods

### 2.1. Animals

C57BL/6 mice were purchased from Charles River. *RAGE* ^−/−^ and* Icam-1* ^−/−^ mice were kindly provided from Peter Nawroth (University Heidelberg) generated as described earlier and backcrossed for at least 15 generations into C57BL/6 background [20, 21]. All mice were maintained at the Central Animal Facility of the University of Heidelberg, Germany. For all experiments, mice were at least 8 weeks of age. All animal experiments were approved by the Animal Care and Use Committee of the Regierungspraesidium Karlsruhe, Germany (AZ G85/11).

### 2.2. Protein C, Cytokines, and Special Reagents

Human protein C concentrate (Ceprotin) was kindly provided from Baxter (Unterschleißheim, Germany), dissolved as indicated in the drug data sheet to an isotonic working solution of 100 U/mL protein C (1 U = 4 *μ*g PC), containing 8 mg/mL human serum albumin for stabilizing reasons. Aliquots were immediately frozen at −80°C and were freshly used for each experiment. Isotonic human serum albumin (Sigma-Aldrich, Taufkirchen, Germany) at 8 mg/mL served as the control solution. PC solution and control solution were further dissolved in normal saline to 200 *μ*L and were administered intraperitoneally. In all experiments, PC was administered at 100 U/kg referring to 400 *μ*g/kg. Human activated protein C (Enzyme Research Laboratory, Swansea, UK) was diluted in normal saline to a working solution of 100 *μ*g/mL and was systemically injected into mice at 24 *μ*g/kg/h, 3 h before intravital microscopy.

In some intravital experiments, fMLP (1 *μ*M,* N-formyl-L-methionyl-L-leucyl-L-phenylalanine; *Sigma, Taufkirchen, Germany) was added to the superfusion buffer to induce additional leukocyte adhesion as described in [22, 23]. In certain experiments, recombinant murine CXCR2 chemokine CXCL-1 (keratinocyte-derived chemokine KC; Peprotech, London, UK) was injected systemically at a dose of 600 ng/mouse. In designated* in vivo* experiments, recombinant murine TNF*α* (R&D Systems, Wiesbaden, Germany) was applied intrascrotally at 500 ng per mouse.

### 2.3. Coagulation Assays

To investigate the plasma APC concentration during PC therapy, mice were first anesthetized by intraperitoneal (i.p.) injection of ketamine (125 mg/kg body weight; Pfizer, Karlsruhe, Germany) and xylazine (12.5 mg/kg body weight; Alverta, Neumuenster, Germany).

Activation of human protein C was analyzed as previously described [24] with some modifications. Briefly, mice were injected with 100 U/kg of human protein C into the tail vein. As positive controls, 50 mU human *α*-thrombin (Hemochrom Diagnostica, Essen, Germany) was additionally injected 10 minutes prior to blood sampling in some experiments. 30 minutes after PC, blood was drawn as a final blood sample by heart puncture into 0.38% sodium citrate and 50 mM benzamidine HCl. Human activated protein C was captured from these plasma samples using the HAPC1555 antibody (kindly provided by C. T. Esmon, Oklahoma Medical Research Foundation, Oklahoma City, USA), which is a highly specific mouse monoclonal antibody against human aPC, developed by standard techniques [25]. Due to the antibodies capacity for capturing from plasma, the direct detection of aPC plasma concentrations is possible [25]. The activity of the captured human PC was determined using a chromogenic substrate (PCa, American Diagnostic) [26].

### 2.4. Intravital Microscopy

As recently reported, we used the cremaster muscle models of trauma- and TNF*α*-induced inflammation [6]. Briefly, after anaesthesia by i.p. injection of ketamine and xylazine (see above) mice were placed on a heating pad to maintain body temperature during surgical preparation and intravital microscopy. Intravital microscopy was conducted on an upright microscope (Leica, Wetzlar, Germany) with a saline immersion objective (SW40/0.75 numerical aperture, Zeiss, Jena, Germany).

### 2.5. Cremaster Muscle Preparation

The surgical preparation of the cremaster muscle was conducted as described previously (trauma-induced inflammation) [6]. Depending on the experimental setting, CXCL-1 chemokine (KC), TNF*α*, or fMLP was applied as stated above in order to induce additional leukocyte adhesion [22]. Microscopic observation of cremaster muscle venules of 20–40 *μ*m diameter was recorded via a CCD camera (CF8/1; Kappa, Gleichen, Germany) on a Panasonic S-VHS recorder. The number of adherent leukocytes (firm adhesion for >30 s) was assessed as adherent cells per mm^2^ vessel surface area. In a separate set of experiments, cremaster muscle whole mounts were obtained as described before [6] and analyzed for intra- and extravascular leukocytes using a Leica DMRB upright microscope and a ×63/0.75 NA oil immersion objective (both from Leica, Wetzlar, Germany).

### 2.6. Leukocyte Recruitment during LPS-Induced Acute Lung Injury (ALI)

To induce acute pulmonary inflammation we adapted the described model of LPS-induced ALI [27]. Briefly, LPS from* E. coli* 0111:B4 (10 *μ*g LPS/50 *μ*L PBS; Sigma-Aldrich, Taufkirchen, Germany) was instilled intratracheally during anaesthetic inhalation of isoflurane (Baxter, Unterschleißheim, Germany) to trigger neutrophil infiltration into the lung. PBS served as negative control. 100 U PC/kg was administered intravenously 0.5 h after LPS installation in WT mice or* RAGE-* or* Icam-1*-deficient mice to dissect the role of RAGE and ICAM-1. Six hours after LPS application mice were anesthetized by i.p. injection as already mentioned, the trachea was cannulated and a bronchoalveolar lavage (BAL) of the right lung was performed using a rinse solution containing PBS and protease inhibitor solution (Protease Inhibitor Cocktail, Sigma-Aldrich, Taufkirchen) to harvest infiltrated cells. For leukocyte differentiation, cells were coated on microscopic slides using a Shandon Cytospin Centrifuge (Thermo Fisher Scientific, Waltham, USA), stained with Giemsa/May Grünwald solution, and analyzed on a Leica DMRB upright microscope and a ×100/0.75 NA oil immersion objective (both from Leica, Wetzlar, Germany).

### 2.7. Flow Cytometry

The expression of Mac-1 and LFA-1 was assessed on murine bone marrow-derived neutrophils using flow cytometry. After red blood cell lysis, leukocytes were treated with PC (5 U/10^6^ leukocytes, 3 h at 37°C). Next, cells were incubated with FITC-conjugated rat anti-Mac-1 mAb M1/70, (eBioscience, San Diego, USA), FITC-conjugated rat anti-LFA-1 mAb M17/4 (eBioscience, San Diego, USA), or respective FITC-conjugated isotype control antibodies (eBioscience, San Diego, USA) to detect anti-Mac-1 and anti-LFA-1 signals. Mac-1 and LFA-1 expression were assessed on 10.000 cells within the neutrophil cluster defined by forward-side scatter analysis using LSRII with DIVA software package (Becton Dickinson, San Jose, USA) and compared to their respective isotype controls.

For flow cytometric analysis of intracellular RAGE and surface ICAM-1 expression of endothelial cells we used cultured murine aortic endothelial cells (MAECs). MAECs were isolated and cultured as described previously [28]. MAECs were grown to near confluence in Greiner 6-well plates (Greiner, Frickenhausen, Germany) and incubated with TNF*α* at 25 ng/mL for 4 h at 37°C. PC pretreatment with 5 U/10^6^ cells was initiated 3 h before TNF*α* stimulation. Cells were then harvested with Accutase (PAA, Cölbe, Germany) and washed with PBS containing 1% bovine serum albumin (BSA). For intracellular RAGE staining, cell permeability was achieved by incubation with PBS containing 0.1% saponin and 5% powdered milk (both from Carl Roth, Karlsruhe, Germany) for 30 min at 4°C. After washing, cells were incubated with FITC-labeled polyclonal rabbit anti-RAGE antibody (BIOSS, Woburn, Massachusetts, USA) or isotype control (Santa Cruz, Heidelberg, Germany) for 45 min on ice. For ICAM-1 staining, cells were incubated with a FITC-conjugated anti-mouse CD54 mAb or isotype control antibody (both from eBioscience, Germany) for 45 min on ice. The antibody signal was detected on 10.000 cells using the four-decade FACS Scan LSRII with DIVA software package.

### 2.8. Model of Lethal Endotoxemia

Lethal endotoxemia was induced by a single i.p. injection of 40 mg/kg LPS (*Escherichia coli*; serotype 055:B5 Sigma, Taufkirchen, Germany) which was reconstituted in 100 *μ*L of sterile PBS, as reported previously [29] with modification. 100 U/kg PC or control solution was administered i.p. at 30 minutes and 8 and 24 hours after LPS challenge. In the first group, survival was observed for 14 days in WT mice and* RAGE-* and* Icam-1*-deficient mice. In the second group, mice were perfused with saline and lungs were harvested 24 h after LPS injection. After fixation in PFA (4%) they were prepared for paraffin-embedded sections. Sections were performed at 3 *μ*m thickness and finally stained with H&E (haematoxylin and eosin staining) for microscopic evaluation.

### 2.9. Statistics

All statistical analyses were performed using Prism 4 (GraphPad, La Jolla, USA). To compare the mortality of PC-treated and control mice during lethal endotoxemia log-rank test of Kaplan-Meier survival distribution was used. Statistical significance between groups and treatments were compared with one-way ANOVA followed by a multiple pairwise comparison test (Newman-Keuls-Test). Statistical significance was set at *P* < 0.05.

## 3. Results

### 3.1. Role of RAGE and ICAM-1 in PC-Induced Inhibition of Leukocyte Adhesion and Transmigration during Trauma-Induced Inflammation

First, we showed that mice injected with zymogen PC were able to significantly activate PC. Nevertheless, PC and thrombin coinjected mice reached even higher levels of PC activation (see Supplementary Figure 1 available online at http://dx.doi.org/10.1155/2014/743678). During trauma-induced inflammation, surgical preparation provokes firm leukocyte arrest mostly mediated via the *β*_2_-integrins LFA-1 and Mac-1 interacting with ICAM-1 and RAGE, respectively [5, 6]. To elucidate the role of RAGE and ICAM-1 in the context of PC-induced inhibition of leukocyte recruitment, leukocyte adhesion in postcapillary venules of surgically prepared cremaster muscles of wild-type (WT),* RAGE* ^−/−^, and* Icam-1* ^−/−^ mice was observed using intravital microscopy.

**Figure 1:**
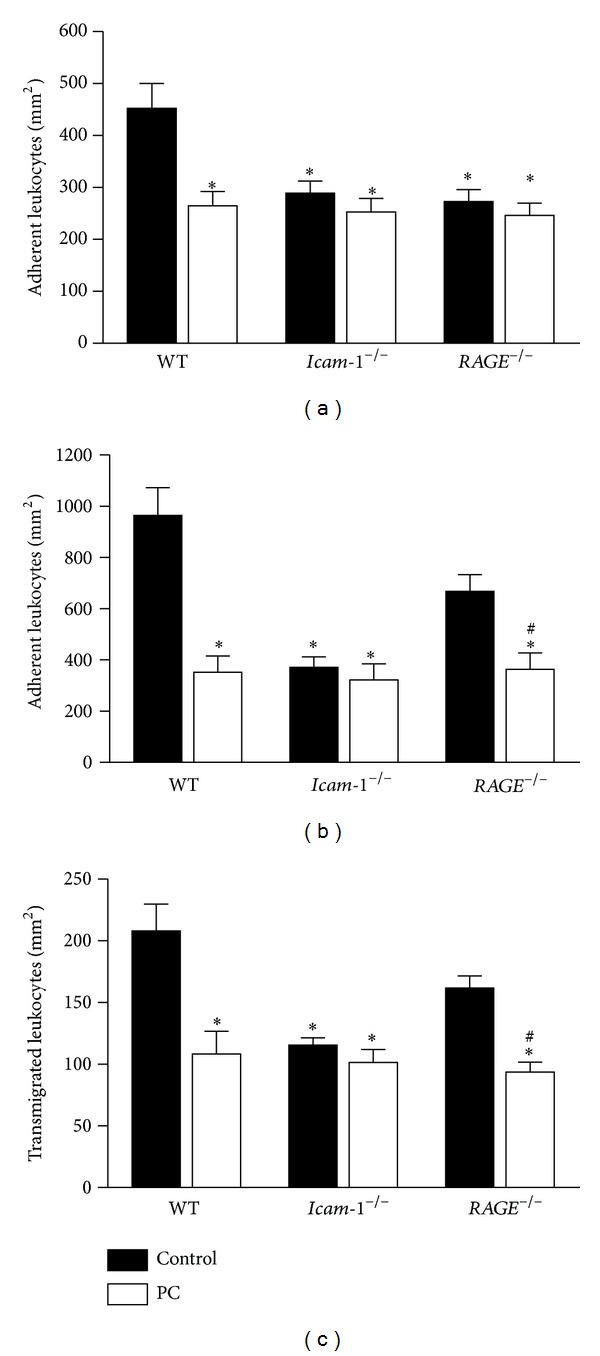
Effect of PC on leukocyte recruitment in wild-type,* RAGE* ^−/−^, and* Icam-1* ^−/−^ mice during trauma-induced inflammation. Leukocyte adhesion (number of adherent cells per mm^2^ of surface area) in cremaster muscle venules of wild-type (WT) control mice,* RAGE* ^−/−^ mice, and* Icam-1* ^−/−^ mice was recorded with and without treatment with PC (100 U/kg, 3 hours) during trauma-induced inflammation (a). Leukocyte adhesion in the same genotypes and treatment groups was shown after additional stimulation with fMLP (superfusion at 1 *μ*M, 15 min) (b). Leukocyte transmigration (per mm^2^ surface area) was analyzed in Giemsa-stained cremaster muscle whole mounts after 15 min fMLP superfusion (1 *μ*M) in the trauma model in WT,* Icam-1* ^−/−^, and* RAGE* ^−/−^ mice with and without PC treatment (100 U/kg, 3 hours) (c). All values are presented as mean ± SEM from three or more mice per group. Significant differences (*P* < 0.05) to WT and* RAGE* ^−/−^ control mice are indicated by the asterisks and pound key, respectively.

Microvascular and hemodynamic parameters did not vary significantly between the treatment groups and genotypes (Supplementary Table 1). In line with our previous findings [11], application of 100 U PC/kg in WT mice resulted in a profoundly inhibited leukocyte adhesion compared to control mice (Figure 1(a)). As observed previously during trauma-induced inflammation [6], both* RAGE* ^−/−^ and* Icam-1* ^−/−^ mice showed significantly reduced numbers of adherent cells compared to WT control mice. Interestingly, PC treatment did not lead to a further inhibition of leukocyte adhesion in both knockout mice. We then asked whether activated PC exerts a stronger anti-inflammatory potential than PC and compared both treatments in* RAGE* ^−/−^ and WT mice. However, aPC and PC showed similar inhibitory effects on leukocyte adhesion in that model (Supplementary Figure 2).

Since the integrin ligands RAGE and ICAM-1 regulate leukocyte recruitment in a stimulus-dependent manner [5], we used further proinflammatory stimuli in the trauma model. First, local superfusion with fMLP (*N-formyl-L-methionyl-L-leucyl-L-phenylalanine*, 1 *μ*M), a potent chemoattractant and integrin activator [5, 22], induced additional leukocyte adhesion in cremaster muscle venules of WT and* RAGE* ^−/−^ mice, but not of* Icam-1* ^−/−^ mice. PC treatment significantly blocked fMLP-stimulated leukocyte adhesion in WT and* RAGE* ^−/−^ mice, while there was no PC effect on leukocyte adhesion in* Icam-1* ^−/−^ mice (Figure 1(b)).

Second, mice were injected with the CXC chemokine CXCL1, also known as keratinocyte-derived chemokine (KC), for triggering additional leukocyte adhesion in exteriorized cremaster muscle venules [5, 22]. PC blocked CXCL1-induced increase of leukocyte adhesion in WT and* RAGE* ^−/−^ mice but not in* Icam-1* ^−/−^ mice (Supplementary Figure 3).

Moreover, we analyzed PC-induced inhibition of leukocyte transmigration. For this purpose, Giemsa staining of whole mounts of fMLP-stimulated cremaster muscles, obtained after the respective intravital microscopic experiment, was performed (Figure 1(c)). In line with leukocyte adhesion, fMLP-induced leukocyte transmigration of WT and* RAGE* ^−/−^ mice was blocked by PC, whereas this finding was not shown in* Icam-1* ^−/−^ mice.

These results indirectly indicate that PC-induced inhibition of leukocyte recruitment may at least in part involve ICAM-1 and RAGE.

### 3.2. Anti-Inflammatory Effects of PC Depend on RAGE, but Not on ICAM-1 during TNF*α*-Induced Inflammation

**Figure 2:**
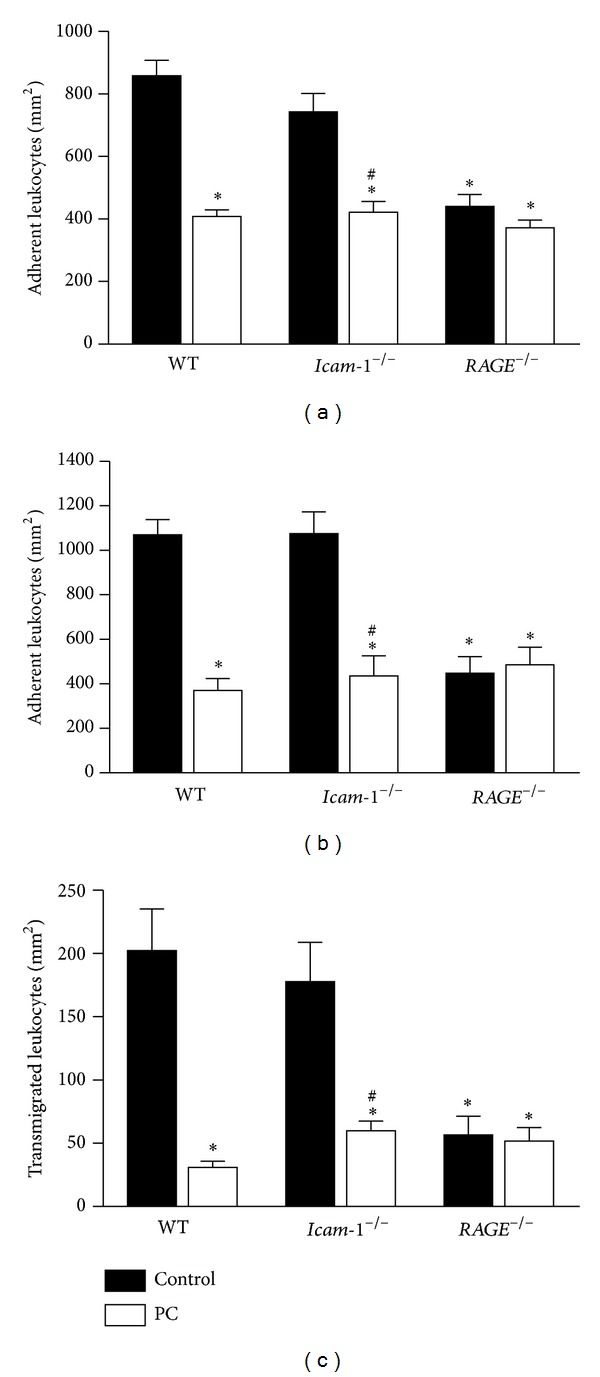
Effect of PC on leukocyte recruitment in wild-type,* RAGE* ^−/−^, and* Icam-1* ^−/−^ mice after TNF*α* stimulation. Leukocyte adhesion (number of adherent cells per mm^2^ of surface area) in 3 h TNF*α*-stimulated (500 ng/mouse) cremaster muscle venules of wild-type (WT) control mice and* Icam-1* ^−/−^ and* RAGE* ^−/−^ mice was documented with and without treatment with PC (100 U/kg, 3 hours) (a). Leukocyte adhesion in the same genotypes and treatment groups was depicted after additional stimulation with fMLP (superfusion at 1 *μ*M, 5 min) (b). Leukocyte transmigration (per mm^2^ surface area) was analyzed in Giemsa-stained cremaster muscle whole mounts after 5 min fMLP superfusion (1 *μ*M) in the TNF*α*-model in WT,* Icam-1* ^−/−^, and* RAGE* ^−/−^ mice with and without PC treatment (100 U/kg, 3 hours) (c). All values are presented as mean ± SEM from three or more mice per group. Significant differences (*P* < 0.05) to WT and* Icam-1* ^−/−^ control mice are indicated by the asterisks and pound key, respectively.

To investigate whether RAGE and ICAM-1 are involved in the mediation of anti-inflammatory properties of PC after TNF*α* stimulation, we measured leukocyte adhesion in 3 h-TNF*α*-treated cremaster muscle venules of WT,* RAGE* ^−/−^, and* Icam-1* ^−/−^ mice with and without PC therapy (100 U/kg 3 h). Consistent with earlier findings [11], PC profoundly blocked leukocyte adhesion in WT mice (Figure 2(a)). Under controlled conditions, leukocyte adhesion of* RAGE*-deficient mice was significantly impaired compared to WT mice, whereas the number of adherent leukocytes in* Icam-1-*deficient mice was comparable to those of WT mice (Figure 2(a)). Similar to WT mice, PC administration significantly reduced leukocyte adhesion in* Icam-1* ^−/−^ mice, while the number of adherent leukocytes was not further decreased in response to PC treatment in* RAGE* ^−/−^ mice.

**Figure 3:**
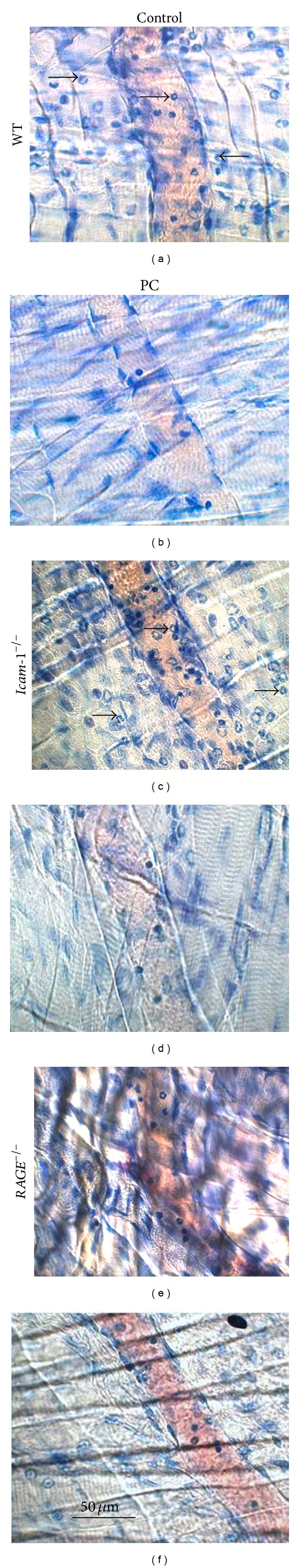
Leukocyte transmigration in PC-treated wild-type,* RAGE* ^−/−^, and* Icam-1* ^−/−^ mice after TNF*α* stimulation. Leukocyte transmigration is illustrated by representative micrographs of Giemsa-stained cremaster muscle whole mounts after 5 min fMLP superfusion (1 *μ*M) in the TNF*α*-model in WT,* Icam-1* ^−/−^, and* RAGE* ^−/−^ mice with and without PC treatment (100 U/kg, 3 hours) (a–f). Reference bar is shown in (c). Arrows point to extravasated leukocytes.

In accordance, experiments with additional superfusion with fMLP (1 *μ*M, 5 min) in 3 h-TNF*α*-stimulated cremaster muscle venules showed a significant PC-induced inhibition of leukocyte adhesion in WT and* Icam-1* ^−/−^ mice, but not in* RAGE* ^−/−^ mice (Figure 2(b)). Next, Giemsa-stained whole mounts of TNF*α*- and fMLP-stimulated cremaster muscles were used to investigate leukocyte transmigration. As depicted in Figures 2(c) and 3(a)–3(f), the genotype-specific effect of PC on leukocyte adhesion did also translate into leukocyte extravasation. Representative micrographs of Giemsa-stained whole mounts illustrate the inhibitory effect of PC on leukocyte transmigration in WT (Figure 3(a) versus Figure 3(b)) and* Icam-1* ^−/−^ mice (Figure 3(c) versus Figure 3(d)) which is not detectable in* RAGE* ^−/−^ mice (Figure 3(e) versus Figure 3(f)). Taken together, these results suggest that after long-term proinflammatory stimulation PC-induced inhibition of leukocyte recruitment is dependent on RAGE, but not on ICAM-1.

### 3.3. RAGE and ICAM-1 Mediate PC-Induced Anti-Inflammatory Effects during Acute Lung Injury

**Figure 4:**
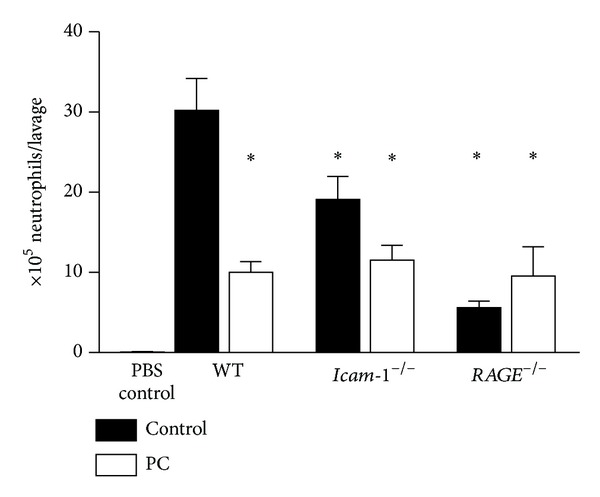
Effect of PC treatment on bronchoalveolar neutrophil emigration during LPS-induced ALI in wild-type,* RAGE* ^−/−^, and* Icam-1* ^−/−^ mice. In a 6-hour model of LPS-induced ALI, the number of intra-alveolar neutrophils obtained by bronchoalveolar lavage was analyzed in wild-type,* Icam-1* ^−/−^, and* RAGE* ^−/−^ mice treated with 100 U/kg PC 30 min after intratracheal LPS instillation (*Escherichia coli* 0111:B4 (10 *μ*g LPS/50 *μ*L PBS)). All values are presented as mean ± SEM from at least three mice per group. Significant differences (*P* < 0.05) to WT control mice are indicated by the asterisks.

To address the question whether RAGE and ICAM-1, which are known to be involved during lung inflammation [30, 31], mediate PC-induced inhibition of leukocyte recruitment in a disease relevant model of acute lung inflammation, we investigated neutrophil emigration during LPS-induced acute lung injury (ALI) in WT,* Icam-1* ^−/−^, and* RAGE* ^−/−^ mice in response to PC. We observed strong neutrophil transmigration into the bronchoalveolar space after LPS instillation. In contrast, the number of bronchoalveolar neutrophils was considerably reduced after PC therapy (Figure 4). In line with previous reports [30, 32], the number of transmigrated neutrophils into acutely inflamed lungs was significantly reduced in* Icam-1* ^−/−^ and* RAGE* ^−/−^ control mice compared to WT control mice. In contrast to* RAGE* ^−/−^ mice, PC treatment did further diminish neutrophil emigration in* Icam-1* ^−/−^ mice (Figure 4), suggesting that the potential of PC to inhibit leukocyte recruitment into the lung is rather related to RAGE than to ICAM-1.

### 3.4. RAGE Mediates PC-Induced Improved Survival during Lethal Endotoxemia

**Figure 5:**
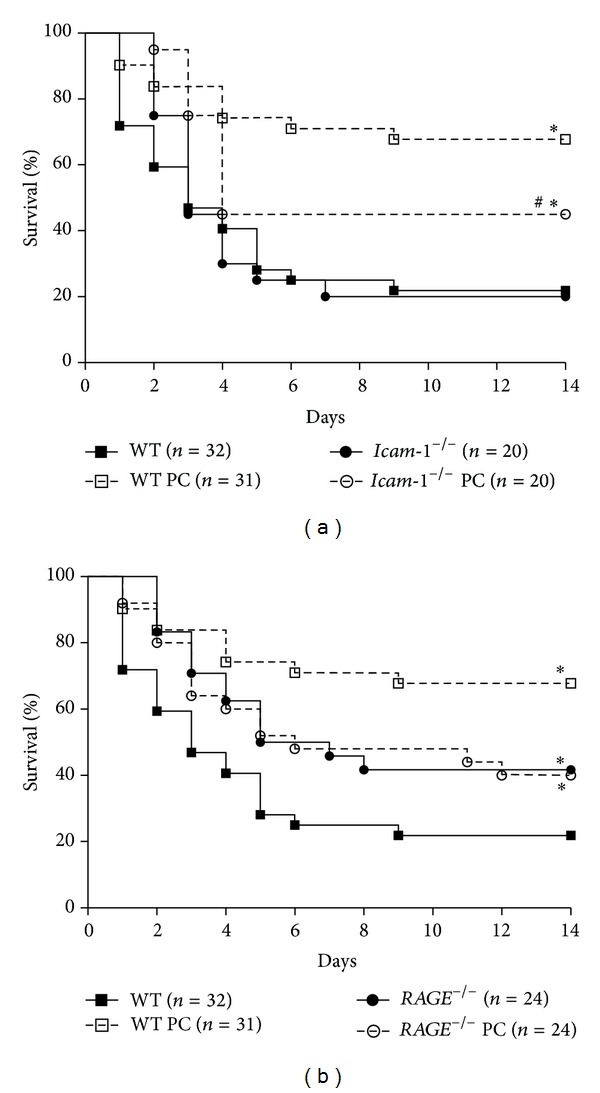
Effect of PC treatment on survival during LPS-induced endotoxemia in wild-type,* RAGE* ^−/−^, and* Icam-1* ^−/−^ mice. Lethal endotoxemia was induced by* Escherichia coli* LPS (serotype 055:B5, 40 mg/kg i.p.) and treated with PC (100 U/kg, i.p.) or human albumin (8 mg/mL) as controls at 0.5, 8, and 24 h after LPS challenge. Survival is shown in Kaplan-Meier plots for the respective treatments in* Icam-1* ^−/−^ mice (a) and* RAGE* ^−/−^ mice (b) and compared to WT mice. Significant differences by log-rank test were set at *P* < 0.05 and indicated by asterisks.

Since PC is known to improve survival during systemic inflammation [11], we asked whether RAGE and ICAM-1 contribute to that improvement of survival. We used an established mouse model of lethal endotoxemia by intraperitoneal injection of* Escherichia coli* LPS (40 mg/kg) followed by treatment with 100 U/kg PC or control solution (human albumin (8 mg/mL)) after 0.5, 8, and 24 hours. PC treatment significantly improved survival in WT and* Icam-1* ^−/−^ mice compared to respective control mice (about 20% versus 70% and 20% versus 45%, resp., Figure 5(a)). Compared to WT mice (20% survival),* RAGE* ^−/−^ mice were protected against LPS-induced endotoxemia (45% survival) (Figure 5(b)). Notably, PC treatment did not further improve survival in* RAGE*-deficient mice (both about 40% survival, Figure 5(b)).

**Figure 6:**
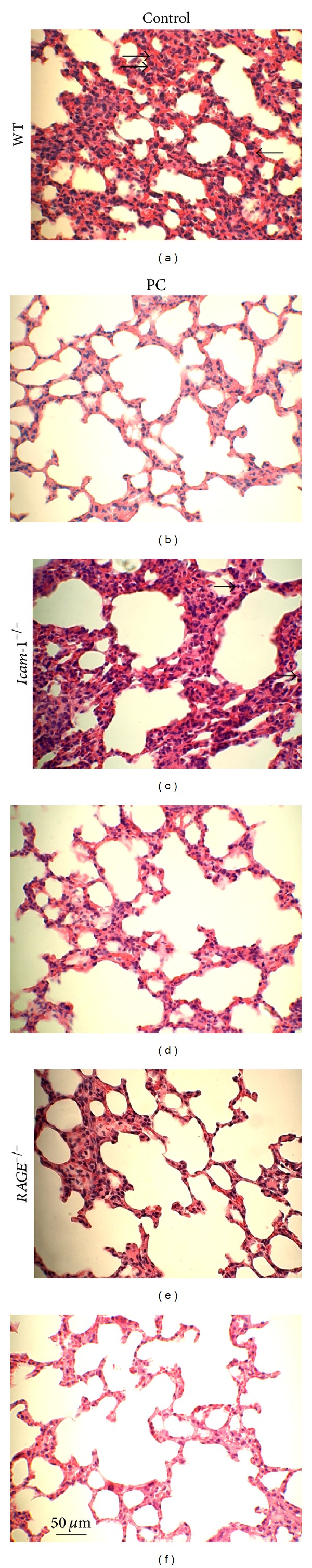
Effect of PC treatment on lung inflammation during LPS-induced endotoxemia in wild-type,* RAGE* ^−/−^, and* Icam-1* ^−/−^ mice. Lethal endotoxemia was induced by* Escherichia coli* LPS (serotype 055:B5, 40 mg/kg i.p.) and treated with PC (100 U/kg, i.p.) or human albumin (8 mg/mL) as controls at 0.5, 8, and 24 h after LPS challenge. Lungs of WT mice (a and b),* Icam-1* ^−/−^ mice (c and d), and* RAGE* ^−/−^ mice (e and f) with and without PC treatment were harvested 24 h after LPS challenge and prepared as 3 *μ*m paraffin-embedded sections for H&E staining. Representative micrographs are shown for at least three mice per group. Arrows indicate infiltrating neutrophils. Reference bar for (a)–(f) is shown in (a) and represents 50 *μ*m.

Next, we determined leukocyte infiltration into the lung after 24 hours of LPS-induced sepsis. PC treatment evidently reduced leukocyte emigration into the lungs of WT mice (Figures 6(a) and 6(b)) and* Icam-1* ^−/−^ mice (Figures 6(c) and 6(d)). In* RAGE* ^−/−^ mice leukocyte infiltration was markedly reduced compared to WT control mice (Figures 6(e) and 6(a)); however, PC treatment did not alter leukocyte emigration into the lung in the absence of RAGE (Figures 6(e) and 6(f)). These results point towards RAGE as an important mediator of anti-inflammatory effects of PC, even during systemic inflammation.

### 3.5. PC Downregulates Intracellular RAGE and Extracellular ICAM-1 of Endothelial Cells, but Not LFA-1 and Mac-1 on Neutrophils

**Figure 7:**
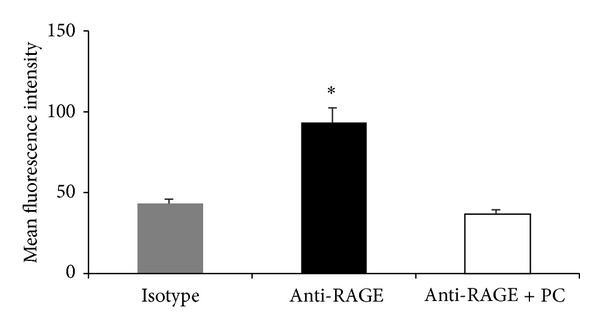
Effect of PC on intracellular RAGE expression in endothelial cells. Intracellular RAGE expression of TNF*α*-stimulated (25 ng TNF*α*/mL, 4 h) endothelial cells was measured by flow cytometry after permeabilization with saponin in response to PC (5 U/10^6^ leukocytes, 3 h) and compared to respective isotype controls. Mean fluorescence intensities are presented as mean ± SEM from at least three mice per group. Significant differences (*P* < 0.05) to isotype and anti-RAGE + PC are indicated by the asterisks.

**Figure 8:**
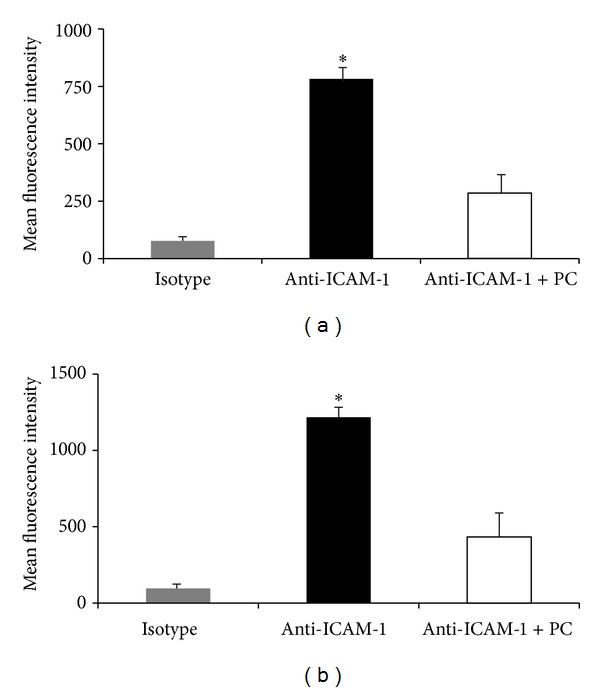
Effect of PC on endothelial ICAM-1 expression. Surface ICAM-1 expression was measured on freshly prepared (a) and TNF*α*-stimulated (25 ng TNF*α*/mL, 4 h) (b) endothelial cells with or without PC preincubation (5 U/10^6^ leukocytes, 3 h) and compared to respective isotype controls. Mean fluorescence intensities are presented as mean ± SEM from at least three mice per group. Significant differences (*P* < 0.05) to isotype and anti-ICAM-1 + PC are indicated by the asterisks.

To explore the underlying mechanisms of the observed PC effects, we investigated the capacity of PC to regulate expression of *β*_2_-integrins of neutrophils and RAGE and ICAM-1 on endothelial cells using flow cytometry. As depicted in Supplementary Figure 4, LFA-1 and Mac-1 expression of neutrophils were not altered in response to PC. However, intracellular accumulation of RAGE was reduced in TNF*α*-stimulated murine aortic endothelial cells (MAEC) after PC treatment (Figure 7). In addition, PC was able to downregulate ICAM-1 expression after PC pretreatment of freshly prepared endothelial cells (Figure 8(a)). These cells were only stimulated by preparation procedures and resemble the surgical preparation of the trauma* in vivo* model. As depicted in Figure 8(b), ICAM-1 expression was similarly downregulated by PC on TNF*α*-stimulated endothelial cells which is surprising in light of our* in vivo *results during TNF*α*-induced inflammation. These results stress the role of PC for the regulation of inflammatory response particularly of the endothelium.

## 4. Discussion

The receptor for advanced glycation end products is a multiligand receptor and is involved in a variety of inflammatory conditions [33]. RAGE activates the proinflammatory NF-*κ*B pathway, but also acts as a ligand for the leukocyte expressed *β*_2_-integrin Mac-1 [5, 6, 15, 19]. Interestingly,* in vivo* experiments using the models of trauma- and TNF*α*-induced inflammation of murine cremaster muscles revealed that RAGE and ICAM-1 act together in mediating leukocyte adhesion in a stimulus-dependent manner [5, 6].

This study provides evidence that RAGE also cooperates with ICAM-1 in mediating anti-inflammatory properties of PC. Established murine inflammation models were used to dissect how the described PC-induced inhibition of leukocyte recruitment [11] is related to RAGE and ICAM-1 [5, 6, 23]. During trauma-induced inflammation, PC requires both RAGE and ICAM-1, which reportedly act in concert in that model [5, 6] in order to inhibit leukocyte adhesion, as there was no detectable effect of PC in* RAGE* ^−/−^ and* Icam-1* ^−/−^ mice. In this model, additional short-term administration of the leukocyte chemoattractant fMLP stimulates ICAM-1 to become more important for mediation of leukocyte adhesion and transmigration [6]. Consequently, ICAM-1 is predominantly involved in PC-induced inhibition of leukocyte recruitment, whereas RAGE is dispensable in that early phase of inflammation.

In contrast, ICAM-1 is no longer a key molecule for leukocyte recruitment during TNF*α*-induced inflammation of cremaster muscles, which is consistent with previous studies [5, 6, 23]. Accordingly, it is not a relevant adhesion molecule targeted by anti-inflammatory functions of PC in that model, which was confirmed by effective PC-induced inhibition of leukocyte recruitment despite the absence of ICAM-1. In context with our present and previous findings that RAGE is crucial for mediation of leukocyte recruitment during TNF*α*-induced inflammation we argue that RAGE might also be critically involved in mediation of the anti-inflammatory potential of PC after TNF*α* stimulation. The finding that PC did not block leukocyte recruitment in* RAGE* ^−/−^ mice after TNF*α* stimulation supports this hypothesis.

Next, we studied whether RAGE and ICAM-1 are linked to the anti-inflammatory PC pathway in more disease relevant mouse models: LPS-induced acute lung injury (LPS-ALI) [34, 35] and LPS-induced lethal endotoxemia [36]. Based on our results during LPS-induced ALI, RAGE and ICAM-1 play a crucial role in leukocyte recruitment during lung inflammation, a finding that has been described in earlier studies [30, 32, 37, 38]. Since PC treatment had no effect on bronchoalveolar neutrophil emigration in* RAGE* ^−/−^ mice but insignificantly reduced the number of neutrophils in BAL of* Icam-1* ^−/−^ mice, we suggest that RAGE, which is abundantly expressed in the lung [31, 39], plays a dominant role for mediating anti-inflammatory effects of PC during acute lung inflammation.

In line with earlier reports [18, 21],* RAGE*-deficient mice showed an improved survival during LPS-induced lethal endotoxemia. However,* ICAM-1* deficiency was not beneficial for survival in our experimental setting, which is in contrast to Xu et al. [20]. The different LPS serotype used in their study may explain the contrary results (0127:B7 versus 055:B5 in our study) [20]. While PC treatment was effective to improve survival of WT mice and* Icam-1*-deficient mice, it failed in* RAGE*-deficient mice. Moreover, mortality during LPS-induced lethal endotoxemia correlated with leukocyte infiltration into the lungs, indicating that the anti-inflammatory potential of PC might be linked to RAGE even during systemic inflammation. However, it should be noted that RAGE is most likely not the only mediator of anti-inflammatory effects of PC in that model.

Taken together, these results provide indirect evidence that RAGE is predominantly involved in the anti-inflammatory PC pathway, particularly during persistent and prolonged inflammatory stimulation, while ICAM-1 is more relevant during brief and mild inflammation. Thus, the findings of this study could stimulate the development of course-specific therapeutic approaches to treat inflammatory diseases involving ICAM-1 and RAGE [15, 16, 33, 40].

To explore how PC interferes with RAGE and ICAM-1, intracellular RAGE and extracellular ICAM-1 expression of endothelial cells were investigated in response to PC. Indeed, the discovered PC-induced downregulation of intracellular RAGE in TNF*α*-stimulated endothelial cells may explain some of the anti-inflammatory effects of PC observed in our* in vivo* inflammation models. Consistent with previous studies [3, 41] we also found that protein C strongly downregulates endothelial ICAM-1 expression. Notably, PC-induced ICAM-1 downregulation was detectable during both mild (cell preparation) and strong (TNF*α*) proinflammatory stimulation. The latter finding is in line with our recent observation [11], but in contrast to our* in vivo* results shows that ICAM-1 plays only a minor role for PC-induced effects during TNF*α*-induced inflammation. However, the technical setup of the* in vitro* and* in vivo* experiments was different.

Further downstream NF-*κ*B and ERK 1/2 mitogen-activated protein kinase (MAPK) might be involved during PC dependent regulation of RAGE and ICAM-1. This hypothesis is supported by the fact that NF-*κ*B and MAPK are linked to both RAGE [42, 43] and ICAM-1 [44–46] on the one hand and are known mediators of endothelial cytoprotective protein C signaling on the other hand [47].

With regard to our* in vivo* results it is important to discuss other potential mechanisms of action of PC like leukocyte-driven effects. As previously reported, activated PC may interfere with the *β*_2_-integrins LFA-1 and Mac-1 [9, 36], which are ligands of ICAM-1 and RAGE, respectively. Although PC did not change Mac-1 and LFA-1 expression of neutrophils, we cannot completely exclude influences of PC on *β*_2_-integrins in our study.

Furthermore, PC might also interact with other RAGE ligands, as described for HMGB1 [48–50]. Likewise, Dinarvand et al. demonstrated in their very recent study that the inorganic polyphosphate induced proinflammatory RAGE signaling can be blocked by activated PC [51]. This study further supports our hypothesis of interplay between the RAGE and PC pathway during inflammation.

In addition to those observations, it is possible that RAGE could be involved in the activation of PC. Consistent with previous findings we showed that the zymogen PC can be activated by mice in our experimental setting [11]. Although similar anti-inflammatory effects of treatment with zymogen PC and activated PC in both WT and RAGE knockout mice might argue against the role of RAGE during PC activation, further thorough investigation of RAGE-dependent PC activation is needed. This, however, is beyond the scope of this paper and should be performed in future studies.

## 5. Conclusion

The anti-inflammatory effect of PC is mediated through and acts on RAGE and ICAM-1 dependent on stimulus. Moreover, the ability of PC to induce its anti- inflammatory effect depends on the role of RAGE in leukocyte recruitment. In turn, PC treatment improved survival during LPS-induced endotoxemia in a RAGE-dependent manner. RAGE and ICAM-1 expression analyses upon PC treatment gave first mechanistic insights. These results may contribute to a better understanding of the immunoregulation by RAGE and the protein C pathway and eventually stimulate further studies.

## Supplementary Material

Activation of human protein C was analyzed as previously described [24] with some modifications. Briefly, mice were injected with 100 U/kg of human protein C into the tail vein. As positive controls, 50 mU human *α*-thrombin (Hemochrom Diagnostica, Essen, Germany) was additionally injected 10 minutes prior to blood sampling in some experiments. 30 minutes after PC, blood was drawn as a final blood sample by heart puncture into 0.38% sodium citrate and 50 mM benzamidin HCl. Human activated protein C was captured from these plasma samples using the HAPC1555 antibody (kindly provided by C. T. Esmon, Oklahoma Medical Research Foundation, Oklahoma City, USA), which is a highly specific mouse monoclonal antibody against human aPC, developed by standard techniques [25]. Due to the antibodies capacity for capturing from plasma, the direct detection of aPC plasma-concentrations is possible [25]. The activity of the captured human PC was determined using a chromogenic substrate (PCa, American Diagnostic) [26].

## References

[1] Rezaie AR (2010). Regulation of the protein C anticoagulant and antiinflammatory pathways. *Current Medicinal Chemistry*.

[2] Jackson CJ, Xue M (2008). Activated protein C—an anticoagulant that does more than stop clots. *International Journal of Biochemistry and Cell Biology*.

[3] Joyce DE, Gelbert L, Ciaccia A, DeHoff B, Grinnell BW (2001). Gene expression profile of antithrombotic protein C defines new mechanisms modulating inflammation and apoptosis. *The Journal of Biological Chemistry*.

[4] Ley K, Laudanna C, Cybulsky MI, Nourshargh S (2007). Getting to the site of inflammation: the leukocyte adhesion cascade updated. *Nature Reviews Immunology*.

[5] Frommhold D, Kamphues A, Dannenberg S (2011). RAGE and ICAM-1 differentially control leukocyte recruitment during acute inflammation in a stimulus-dependent manner. *BMC Immunology*.

[6] Frommhold D, Kamphues A, Hepper I (2010). RAGE and ICAM-1 cooperate in mediating leukocyte recruitment during acute inflammation in vivo. *Blood*.

[7] Springer TA (1995). Traffic signals on endothelium for lymphocyte recirculation and leukocyte emigration. *Annual Review of Physiology*.

[8] Sturn DH, Kaneider NC, Feistritzer C, Djanani A, Fukudome K, Wiedermann CJ (2003). Expression and function of the endothelial protein C receptor in human neutrophils. *Blood*.

[9] Elphick GF, Sarangi PP, Hyun Y-M (2009). Recombinant human activated protein C inhibits integrin-mediated neutrophil migration. *Blood*.

[10] Messaris E, Betrosian AP, Memos N (2010). Administration of human protein C improves survival in an experimental model of sepsis. *Critical Care Medicine*.

[11] Frommhold D, Tschada J, Braach N (2011). Protein C concentrate controls leukocyte recruitment during inflammation and improves survival during endotoxemia after efficient in vivo activation. *The American Journal of Pathology*.

[12] Kerschen EJ, Fernandez JA, Cooley BC (2007). Endotoxemia and sepsis mortality reduction by non-anticoagulant-activated protein C. *The Journal of Experimental Medicine*.

[13] Kau JH, Shih YL, Lien TS (2012). Activated protein C ameliorates *Bacillus anthracis* lethal toxin-induced lethal pathogenesis in rats. *Journal of Biomedical Science*.

[14] Sopel MJ, Rosin NL, Falkenham AG (2012). Treatment with activated protein C (aPC) is protective during the development of myocardial fibrosis: an angiotensin II infusion model in mice. *PLoS ONE*.

[15] van Zoelen MAD, Achouiti A, van der Poll T (2011). RAGE during infectious diseases. *Frontiers in Bioscience*.

[16] Lange-Sperandio B, Sperandio M, Nawroth P, Bierhaus A (2007). RAGE signaling in cell adhesion and inflammation. *Current Pediatric Reviews*.

[17] Liliensiek B, Weigand MA, Bierhaus A (2004). Receptor for advanced glycation end products (RAGE) regulates sepsis but not the adaptive immune response. *The Journal of Clinical Investigation*.

[18] Yamamoto Y, Harashima A, Saito H (2011). Septic shock is associated with receptor for advanced glycation end products ligation of LPS. *The Journal of Immunology*.

[19] Chavakis T, Bierhaus A, Al-Fakhri N (2003). The pattern recognition receptor (RAGE) is a counterreceptor for leukocyte integrins: a novel pathway for inflammatory cell recruitment. *The Journal of Experimental Medicine*.

[20] Xu H, Gonzalo JA, St. Pierre Y (1994). Leukocytosis and resistance to septic shock in intercellular adhesion molecule 1-deficient mice. *The Journal of Experimental Medicine*.

[21] Liliensiek B, Weigand MA, Bierhaus A (2004). Receptor for advanced glycation end products (RAGE) regulates sepsis but not the adaptive immune response. *The Journal of Clinical Investigation*.

[22] Frommhold D, Mannigel I, Schymeinsky J (2007). Spleen tyrosine kinase Syk is critical for sustained leukocyte adhesion during inflammation in vivo. *BMC Immunology*.

[23] Foy DS, Ley K (2000). Intercellular adhesion molecule-1 is required for chemoattractant-induced leukocyte adhesion in resting, but not inflamed, venules in vivo. *Microvascular Research*.

[24] Weiler H, Lindner V, Kerlin B (2001). Characterization of a mouse model for thrombomodulin deficiency. *Arteriosclerosis, Thrombosis, and Vascular Biology*.

[25] Liaw PCY, Ferrell G, Esmon CT (2003). A monoclonal antibody against activated protein C allows rapid detection of activated protein C in plasma and reveals a calcium ion dependent epitope involved in factor Va inactivation. *Journal of Thrombosis and Haemostasis*.

[26] Isermann B, Vinnikov IA, Madhusudhan T (2007). Activated protein C protects against diabetic nephropathy by inhibiting endothelial and podocyte apoptosis. *Nature Medicine*.

[27] Reutershan J, Basit A, Galkina EV, Ley K (2005). Sequential recruitment of neutrophils into lung and bronchoalveolar lavage fluid in LPS-induced acute lung injury. *The American Journal of Physiology: Lung Cellular and Molecular Physiology*.

[28] Kobayashi M, Inoue K, Warabi E, Minami T, Kodama T (2005). A simple method of isolating mouse aortic endothelial cells. *Journal of Atherosclerosis and Thrombosis*.

[29] Kerschen EJ, Fernandez JA, Cooley BC (2007). Endotoxemia and sepsis mortality reduction by non-anticoagulant-activated protein C. *The Journal of Experimental Medicine*.

[30] Basit A, Reutershan J, Morris MA, Solga M, Rose CE, Ley K (2006). ICAM-1 and LFA-1 play critical roles in LPS-induced neutrophil recruitment into the alveolar space. *The American Journal of Physiology: Lung Cellular and Molecular Physiology*.

[31] Uchida T, Shirasawa M, Ware LB (2006). Receptor for advanced glycation end-products is a marker of type I cell injury in acute lung injury. *American Journal of Respiratory and Critical Care Medicine*.

[32] Reynolds PR, Schmitt RE, Kasteler SD (2010). Receptors for advanced glycation end-products targeting protect against hyperoxia-induced lung injury in mice. *American Journal of Respiratory Cell and Molecular Biology*.

[33] Bierhaus A, Nawroth PP (2009). Multiple levels of regulation determine the role of the receptor for AGE (RAGE) as common soil in inflammation, immune responses and diabetes mellitus and its complications. *Diabetologia*.

[34] Riewald M, Petrovan RJ, Donner A, Mueller BM, Ruf W (2002). Activation of endothelial cell protease activated receptor 1 by the protein C pathway. *Science*.

[35] Weiler H (2010). Regulation of inflammation by the protein C system. *Critical Care Medicine*.

[36] Cao C, Gao Y, Li Y, Antalis TM, Castellino FJ, Zhang L (2010). The efficacy of activated protein C in murine endotoxemia is dependent on integrin CD11b. *The Journal of Clinical Investigation*.

[37] Ramsgaard L, Englert JM, Manni ML (2011). Lack of the receptor for advanced glycation end-products attenuates *E. coli* pneumonia in mice. *PLoS ONE*.

[38] Su X, Looney MR, Gupta N, Matthay MA (2009). Receptor for advanced glycation end-products (RAGE) is an indicator of direct lung injury in models of experimental lung injury. *American Journal of Physiology - Lung Cellular and Molecular Physiology*.

[39] Brett J, Schmidt AM, Shi Du Yan SDY (1993). Survey of the distribution of a newly characterized receptor for advanced glycation end products in tissues. *American Journal of Pathology*.

[40] Buschmann K, Koch L, Braach N (2012). CXCL1-triggered interaction of LFA1 and ICAM1 control glucose-induced leukocyte recruitment during inflammation *in vivo*. *Mediators of Inflammation*.

[41] Scaldaferri F, Sans M, Vetrano S (2007). Crucial role of the protein C pathway in governing microvascular inflammation in inflammatory bowel disease. *The Journal of Clinical Investigation*.

[42] Ishihara K, Tsutsumi K, Kawane S, Nakajima M, Kasaoka T (2003). The receptor for advanced glycation end-products (RAGE) directly binds to ERK by a D-domain-like docking site. *FEBS Letters*.

[43] Xu X, Chen H, Zhu X (2013). S100A9 promotes human lung fibroblast cells activation through receptor for advanced glycation end-product-mediated extracellular-regulated kinase 1/2, mitogen-activated protein-kinase and nuclear factor-kappaB-dependent pathways. *Clinical and Expirimental Immunology*.

[44] Joyce DE, Gelbert L, Ciaccia A, DeHoff B, Grinnell BW (2001). Gene expression profile of antithrombotic protein C defines new mechanisms modulating inflammation and apoptosis. *The Journal of Biological Chemistry*.

[45] Yang L, Froio RM, Sciuto TE, Dvorak AM, Alon R, Luscinskas FW (2005). ICAM-1 regulates neutrophil adhesion and transcellular migration of TNF-*α*-activated vascular endothelium under flow. *Blood*.

[46] Wang J, Dong S (2012). ICAM-1 and IL-8 are expressed by DEHP and suppressed by curcumin through ERK and p38 MAPK in human umbilical vein endothelial cells. *Inflammation*.

[47] Guitton C, Cottereau A, Gérard N (2011). Protective cross talk between activated protein C and TNF signaling in vascular endothelial cells: Implication of EPCR, noncanonical NF-*κ*B, and ERK1/2 MAP kinases. *The American Journal of Physiology: Cell Physiology*.

[48] Abeyama K, Stern DM, Ito Y (2005). The N-terminal domain of thrombomodulin sequesters high-mobility group-B1 protein, a novel antiinflammatory mechanism. *The Journal of Clinical Investigation*.

[49] Luo Y, Li S-J, Yang J, Qiu Y-Z, Chen F-P (2013). HMGB1 induces an inflammatory response in endothelial cells via the RAGE-dependent endoplasmic reticulum stress pathway. *Biochemical and Biophysical Research Communications*.

[50] Bae J-S, Rezaie AR (2011). Activated protein C inhibits high mobility group box 1 signaling in endothelial cells. *Blood*.

[51] Dinarvand P, Hassanian SM, Qureshi SH (2014). Polyphosphate amplifies proinflammatory responses of nuclear proteins through interaction with receptor for advanced glycation end products and P2Y1 purinergic receptor. *Blood*.

