# Supplementary material for: Anti-Inflammatory Functions of Protein C Require RAGE and ICAM-1 in a Stimulus-Dependent Manner
Source: Mediators Inflamm. 2014 May 4;2014:743678. doi: 10.1155/2014/743678 (PMC4024424; doi:10.1155/2014/743678)
Supplement: Supplementary file 1 — Activation of human protein C was analyzed as previously described [24] with some modifications. Briefly, mice were injected with 100 U/kg of human protein C into the tail vein. As positive controls, 50 mU human α-thrombin (Hemochrom Diagnostica, Essen, Germany) was additionally injected 10 minutes prior to blood sampling in some experiments. 30 minutes after PC, blood was drawn as a final blood sample by heart puncture into 0.38% sodium citrate and 50 mM benzamidin HCl. Human activated protein C was captured from these plasma samples using the HAPC1555 antibody (kindly provided by C. T. Esmon, Oklahoma Medical Research Foundation, Oklahoma City, USA), which is a highly specific mouse monoclonal antibody against human aPC, developed by standard techniques [25]. Due to the antibodies capacity for capturing from plasma, the direct detection of aPC plasma-concentrations is possible [25]. The activity of the captured human PC was determined using a chromogenic substrate (PCa, American Diagnostic) [26]. [file 743678.f1.zip › 743678.f1/mat.743678.v2.pdf]

Supplemental Material

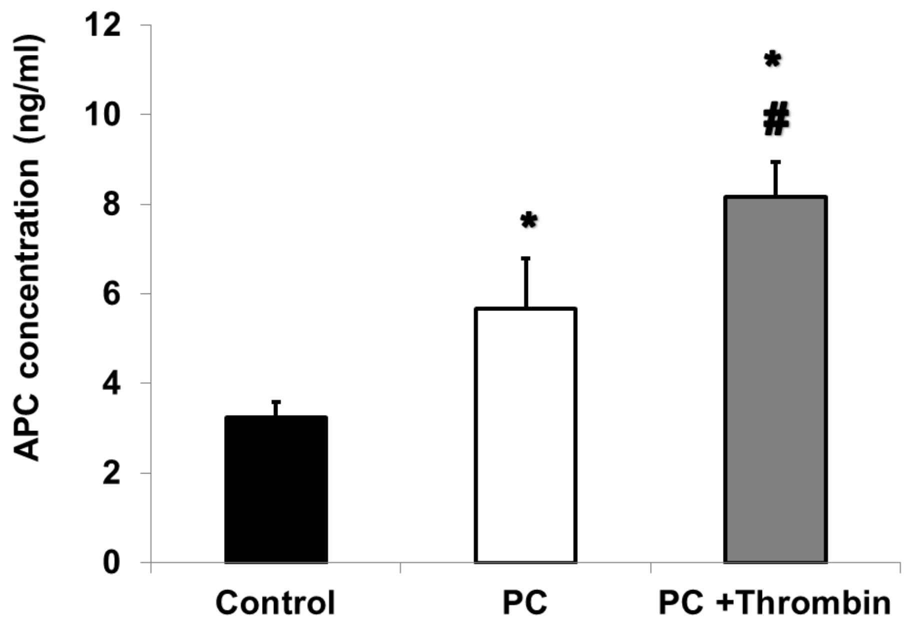

Supplemental Figure 1. *In vivo* activation of PC. TNFα-stimulated WT mice were treated with 100 U/kg PC for 30 min and compared to control mice. In some experiments, additionally administered human α-thrombin enhanced PC activation and served as positive control (PC + Thrombin). All values are presented as mean ± SEM from at least three mice per group. Significant differences ($P < 0.05$) to control mice in are indicated by the asterisks and between PC & Thrombin and PC treated mice by the pound key.

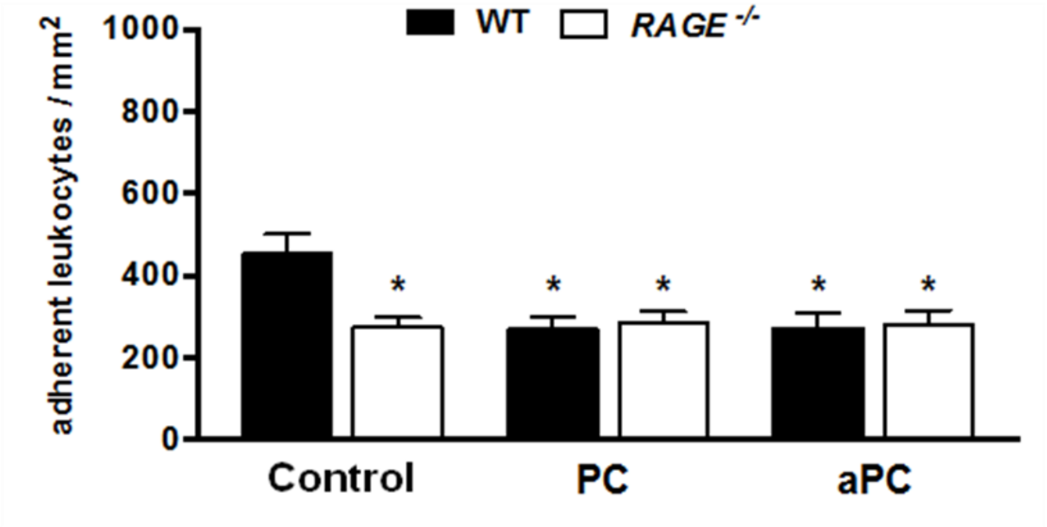

Supplemental Figure 2. Comparison of PC and aPC-induced effects on leukocyte adhesion in wild-type (WT) and *RAGE*$^{-/-}$ mice. Leukocyte adhesion (number of adherent cells per mm^2 of surface area) in cremaster muscle venules of wild-type (WT) control mice and *RAGE*$^{-/-}$ mice treated with PC (100 U/kg, 3 hours) or aPC (24µg/kg/h, 3 hours) during trauma-induced inflammation. All values are presented as mean ± SEM from three or more mice per group. Significant differences ($P < 0.05$) to WT control mice are indicated by the asterisks.

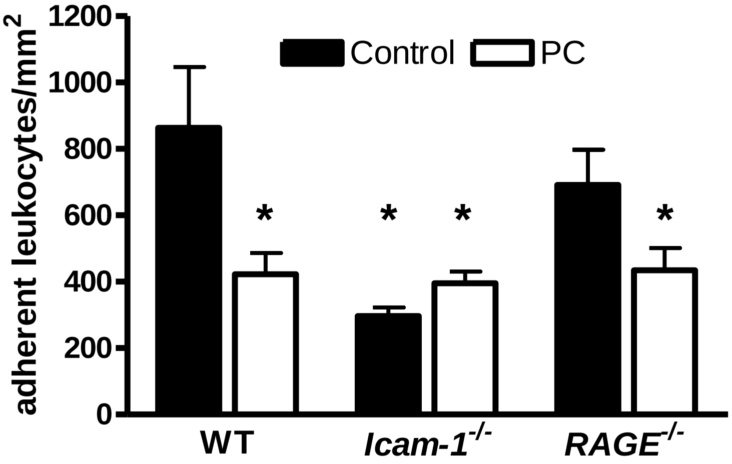

Supplemental Figure 3. Effect of PC on CXCL1-induced leukocyte adhesion in wild-type (WT), *RAGE*$^{-/-}$ - and *Icam-1*$^{-/-}$ mice. Leukocyte adhesion (number of adherent cells per mm^2 of surface area) in cremaster muscle venules after systemic administration of 600 ng CXCL1 (KC) of wild-type (WT) control mice, *RAGE*$^{-/-}$ mice and *Icam-1*$^{-/-}$ mice treated with and without PC (100 U/kg, 3 hours) during trauma-induced inflammation. All values are presented as mean ± SEM from three or more mice per group. Significant differences ($P < 0.05$) to WT control mice are indicated by the asterisks.

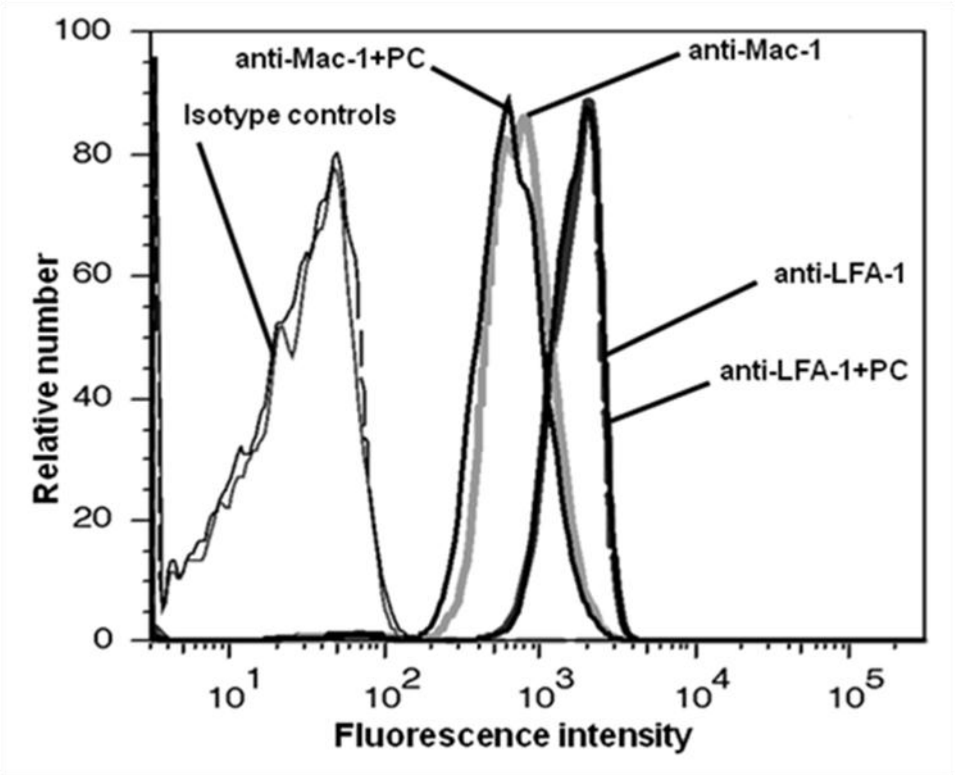

Supplemental Figure 4: Effect of PC on expression of LFA-1 and Mac-1 on neutrophils. Surface expression of LFA-1 and Mac-1 on bone marrow-derived neutrophils with or without PC pre-incubation (5 U per 10^6 leukocytes/ml, 3 h at 37°C) was compared to respective isotype controls. Representative histograms are shown from 3 separate experiments.

Supplemental Table 1: Hemodynamic parameters during intravital microscopic experiments

	Mice	*Venules*	*Diameter*	*Centerline Velocity*	*Wall Shear Rate*	*Systemic Leukocyte Counts*
	N	n	(µm)	(µm/s)	(s-1)	(/µl)
Trauma-induced inflammation Genotype/Treatment (3 hours 100 U/kg PC)						
WT	10	20	30 ± 1	2500 ± 100	1900 ± 100	7300 ± 300
WT PC	10	20	31 ± 1	2700 ± 100	2200 ± 100	6200 ± 400
Icam-1$^{-/-}$	11	25	30 ± 1	2300 ± 100	2000 ± 100	8000 ± 1000
Icam-1$^{-/-}$ PC	13	32	31 ± 1	2300 ± 100	1800 ± 100	9500 ± 500
RAGE$^{-/-}$	12	30	30 ± 1	2500 ± 100	2100 ± 100	7000 ± 700
RAGE$^{-/-}$ PC	11	25	29 ± 1	2400 ± 100	2000± 100	7400 ± 900
			n.s.	n.s.	n.s.	n.s.
TNFα-induced inflammation Genotype/Treatment (3 hours 100 U/kg PC) pre and post fMLP						
WT	10	58	29 ± 1	2000 ± 100	1900 ± 100	4100 ± 500
WT PC	11	60	28 ± 1	2100± 100	1700 ± 100	3900 ± 200
Icam-1$^{-/-}$	7	40	31 ± 1	1900 ± 100	1700 ± 100	4400 ± 500
Icam-1$^{-/-}$ PC	11	44	31 ± 1	2000 ± 50	1800 ± 100	4200 ± 300
RAGE$^{-/-}$	11	49	29 ± 1	2200 ± 100	2000 ± 100	3600 ± 200
RAGE$^{-/-}$ PC	12	62	29 ± 1	2200 ± 100	1900 ± 100	3800 ± 200
			n.s.	n.s.	n.s	n.s.

Vessel diameter, centerline velocity and wall shear rate of surgically prepared cremaster muscle venules (Trauma) and tumor necrosis factorα (TNFα)-stimulated (pre and post fMLP-superfusion) cremaster muscle venules of wild-type (WT), *Icam1*$^{-/-}$ - and *RAGE*$^{-/-}$ mice with and without PC are presented as mean ± SEM.

n.s., not significant.
